# Conceptualization of population-specific human functional immune-genomics projects to identify factors that contribute to variability in immune and infectious diseases

**DOI:** 10.1016/j.heliyon.2021.e06755

**Published:** 2021-04-13

**Authors:** Collins K. Boahen, Leo A.B. Joosten, Mihai G. Netea, Vinod Kumar

**Affiliations:** a Department of Internal Medicine and Radboud Center for Infectious Diseases (RCI), Radboud University Medical Center, Nijmegen, 6525 HP, the Netherlands; b Department for Genomics & Immunoregulation, Life and Medical Sciences Institute (LIMES), University of Bonn, Germany; c University of Groningen, University Medical Center Groningen, Department of Genetics, Groningen, 9700 RB, the Netherlands; d Nitte (Deemed to be University), Nitte University Centre for Science Education and Research (NUCSER), Medical Sciences Complex, Deralakatte, Mangalore, 575018, India

**Keywords:** Human functional genomics projects (HFGPs), Omics approaches, Immune response, Cytokines

## Abstract

The human immune system presents remarkable inter-individual variability in response to pathogens or perturbations. Recent high-throughput technologies have enabled the identification of both heritable and non-heritable determinants of immune response variation between individuals. In this review, we summarize the advances made through the Human Functional Genomics Projects (HFGPs), challenges and the need for more refined strategies. Inter-individual variability in stimulation-induced cytokine responses is influenced in part by age, gender, seasonality, and gut microbiome. Host genetic regulators especially single nucleotide polymorphisms in multiple immune gene loci, particularly the TLR1-TLR6-TLR10 locus, have been identified using individuals of predominantly European descent. However, transferability of such findings to other populations is challenging. We are beginning to incorporate diverse population cohorts and leverage multi-omics approaches at single cell level to bridge the current knowledge gap. We believe that such an approach presents the opportunities to comprehensively assess both genetic and environmental factors driving variation seen in immune response phenotype and a better understanding of the molecular and biological mechanisms involved.

## 1 Introduction

The last decade has witnessed a series of cohort-based studies that emphasized the role of both host and environmental factors such as diet and lifestyle in determining human phenotypes in health and disease conditions. It also has become clear that the interaction of host genetics and environmental factors (among which pathogens causing infections) is extremely dynamic, cell-type specific, and varies between individuals. Understanding the impact of these complex interactions on the function of the cell types in individual-specific manner is crucial to delineate the molecular basis of variability in phenotypes. However, capturing the impact of host genetic, pathogen and environmental factors on all the cellular and biochemical responses that affect phenotypes by any single omics approach is challenging. To tackle these challenges, we [1] and others [2] have conceptualized the human functional genomic projects (HFGPs), in which the main goal is to comprehensively characterize and understand inter-individual variation of human phenotypes by combining ‘omics’ technologies and system biology strategies with in-depth functional phenotyping of the biochemical responses in healthy and diseased individuals. Although this strategy has been applied to study several complex diseases in humans, most progress has been made in immune and infectious diseases. In this review, we would like to highlight some of the recent and important biological insights gained from such studies. We also try and identify limitations of the current strategies and future challenges.

## 2 Context-dependent regulation of molecular responses triggered initiation of HFGPs

Most common human diseases such as diabetes, autoimmune diseases, cancer and infectious diseases are complex phenotypes where multiple genetic and environmental factors are involved. Genome-wide association studies (GWAS) have been commonly applied to identify genetic factors and have been extremely successful in identifying thousands of genetic loci that are significantly associated with hundreds of such common human diseases [3, 4]. However, pinpointing causal genes and causal single nucleotide polymorphisms (SNPs) from these loci remained a bottleneck to obtain further biological insights [5]. To tackle this challenge, several expression-quantitative trait loci (eQTL) mapping studies, mainly using blood tissue, were conducted [6]. Although, these studies helped to establish the link between potential causal genes and disease SNPs at several loci, for more than 50% of the loci no eQTL genes were found [7]. Traditionally, these eQTL studies made use of blood cells from healthy volunteers to identify association between SNPs and gene expression. However, these studies failed to capture the impact of interaction between environmental triggers and genetic variation.

We know from many gene expression studies that hundreds to thousands of genes show differential expression depending on external stimulation. However, only in the beginning of this decade researchers started asking the question whether genetic variants also influence the expression of genes upon stimulation. This led to several stimulation specific eQTL studies, in which eQTL mappings were performed upon stimulation of different immune cells with microbial antigens. These studies not only helped to reveal novel context-specific or response eQTLs [8, 9, 10], but also suggested that identifying such response eQTLs can help in identifying disease genes. For example, it was shown that such response eQTLs to *Mycobacterium tuberculosis* were more likely to be genetically associated with the pulmonary TB disease [8]. However, these studies assessed only RNA levels and did not investigate the changes in other molecular responses such as inflammatory cytokines or metabolites.

During the last decade, we studied systemic candidiasis to identify *Candida*-specific host defense mechanisms. For this, we performed comprehensive analysis of transcriptional responses to *Candida albicans* and identified type 1 interferon pathway as one of the important Candida-specific host defence pathways [11]. Interestingly, SNPs in type I interferon genes affected Candida-induced cytokine production and were associated with susceptibility to systemic candidiasis. To further validate this observation, we profiled cytokines produced by peripheral blood mononuclear cells from 197 individuals of European origin from the 200 Functional Genomics (200FG) cohort in the Human Functional Genomics Project [12] in response to *Candida* and identified SNPs that affect cytokines levels through cytokine quantitative trait loci (cQTL) analysis. Interestingly, a cQTL (rs11141235) of Candida-induced IL-6 at the *NAA35*-*GOLM1* locus was associated with susceptibility to candidemia. These studies strongly suggested the causal role of genetic variants in infectious diseases that affect not only gene expression but also cytokine responses in a context-specific manner. These were some of the findings from earlier studies together with development in high throughput ‘omics’ technologies that led to the conceptualization of HFGPs. In the following sections we will discuss some of the insights gained by such studies as well as the application of appropriate ‘omics’ technologies.

Until the inception of the HFGPs, there have been no comprehensive studies in humans to investigate the basis for inter-individual variability in cytokine responses after stimulation with pathogens. Although results from this pilot study of 200 Functional Genomics (200FG) cohort from our group [12] indicated the feasibility of identifying disease relevant SNPs and genes using this approach, the study itself had several limitations. These were mainly the small sample size, and the availability of only cytokine data and genetic data. To tackle some of these challenges the 500 Functional Genomics (500FG) study was initiated.

## 3 Insights gained from 500 functional genomics project

The 500FG comprises of healthy adults' volunteers recruited from Western Europe (the Netherlands) whose DNA, microbiome, urine, serum and plasma samples are available. By profiling comprehensive molecular, immunological and lifestyle parameters in the 500FG cohort, we have applied functional genomics approach [13] to understand major factors that drives inter-individual variability in cytokine response to several infectious and metabolic triggers.

In the first study within 500FG, we [14] focused on the effect of environmental and non-genetic host factors (such as age, sex and seasonality) on cytokine production after stimulation with 19 bacterial, fungal, viral and non-microbial metabolic stimuli. We identified a strong impact of non-genetic host factors such as age and sex; and additional novel finding being seasonality effect on cytokine responses. For example, in examining gender effects, the production of proinflammatory cytokines released from monocytes was higher in men after stimulation with diverse stimuli and the role of circulating hormone levels failed to explain this gender differences. A second study [13] assessed the impact of genetic variation on cytokine production and elucidated a strong impact genetic heritability with varying effects on different cytokines in whole blood, peripheral blood mononuclear cells and macrophages. The strongest association among the detected 17 (cQTLs) was at the TLR1-TLR6-TLR10 locus, which was also found to be under strong evolutionary selection [15]. Strikingly, no genome-wide significant cQTLs were identified for production of cytokines in macrophages, suggesting that the process of in vitro macrophage differentiation may neutralize the strong differences in cytokine production between individuals. Another fascinating observation from this study was that many cQTLs overlap with loci previously associated with human diseases, particularly immune-mediated diseases and infectious diseases. Similarly, Aguirre-Gamboa et al. (2016) by investigating environmental and genetic factors on immune cell populations in peripheral blood and immunoglobulin levels, identified eight independent genomic loci associated with cell count variation and these QTLs overlapped with GWAS SNPs already known to increase susceptibility to immune-mediated diseases [16].

These findings indicated that performing stimulation studies using disease relevant antigens or triggers may help understand disease mechanisms. For e.g. to understand the factors that contribute to heterogeneity in the symptoms and outcome of Lyme disease, another study [17] leveraged functional genomics approach to Borrelia-induced cytokine response in humans. By conducting genetic, transcriptomic, and functional validation experiments this study revealed an important role for HIF-1α-mediated glycolysis in regulating the cytokine response to Borrelia. Furthermore, HIF-1α pathway activation and increase in glycolysis-derived lactate was also confirmed in Lyme disease patients, which leads to different symptoms and outcome of Lyme disease. Likewise, an extensive GWAS was performed to examine the effect of genetic variants on candidemia susceptibility [18]. By integrating candidemia susceptibility SNPs with *Candida*-induced cQTLs, the researchers demonstrate that up to 35% of the susceptibility loci influence in vitro cytokine response upon *Candida* stimulations, further illustrating how stimulation studies may facilitate our understanding of disease pathogenesis.

The role of host microbiome in health and disease is gaining much needed attention. The impact of gut microbiome on cytokine production has already been discussed elsewhere [19, 20, 21]. In the context of gut microbiome, another study from 500FG cohort investigated how differences in composition and function of gut microbial communities lead to inter-individual variation in cytokines responsiveness to microbial stimulations [22]. Here, the researchers measured both monocyte and lymphocyte-derived cytokines response to three bacteria (LPS, *B. fragilis*, and *S. aureus*) and two fungal (*C. albicans* conidia and hyphae) stimulations. As a result of the significant inter-individual variation in both cytokine responses and gut microbial profiles, the researchers performed correlation analysis between these phenotypes which led to the identification of three microbiome-cytokine interaction patterns: stimulus specific (where gut microbial features were correlated with most cytokines, but related to a particular stimulation), cytokine specific (associations were independent of the type of stimulations), and cytokine and stimulus specific patterns (in this case, gut microbial features affected a particular cytokine response and related to a specific stimulation). The authors further demonstrated that specific host-microbial interactions can lead to modulation of specific metabolic pathways, which in turn may affect the cytokine production. For e.g. in case of TNF-α and IFN-γ production in response to *Candida albicans*, it was shown that the levels of these cytokines were significantly correlated with the microbial metabolic pathways, namely palmitoleic acid metabolism and tryptophan degradation to tryptophol. Interestingly, these effects were specific to monocyte derived cytokines but not T-cell derived cytokines. The biological mechanism responsible for this specificity remain to be investigated.

Although overall, we observed significant impact of genetics, microbiome and non-genetic factors on cytokine responses, it is crucial to tease apart the proportion of variance explained by each of these factors to the inter-individual variability in cytokine responses. Therefore, the quantitative contribution of immunological and molecular profiles to stimulated cytokine production was systematically assessed [23]. The authors found that genetic variation (SNPs) explained most of the variance (R^2^ = 0.18), while gut microbiome, immune cell counts, circulating metabolites and seasonality showed moderate effects (avg.adj R^2^ = 0.061, 0.057, 0.041 and 0.041, respectively). Overall, the afore-mentioned studies pinpoint the enormous advancement made towards our quest to understand the basis of inter-individual differences in cytokine responses upon stimulation. Although, these studies (summarized in Table 1) focused mainly on cytokine production, the comprehensive understanding of the immune landscape variation between individuals requires the consideration of other molecular phenotypes.

**Table 1:** Studies under 500FG investigating determinants of cytokine response variability.

Author (Year)	Factor(s) analyzed	Main Findings
Ter Horst et al. (2016)	Environmental and non-genetic host factors such as age, sex, BMI, oral contraceptive, smoking, vitamin D and seasonality	1. Non-genetic host factors such as age and sex have strong influence on cytokine production, with elevated levels of IL-Ra and IL6- in older individuals 2. Cytokine responses are dependent on annual seasonality 3. Limited effect of vitamin D on cytokine production
Li et al. (2016)	Host genome/genetic variation	1. Strong impact of heritability on cytokine response after bacterial, fungal, viral, non-microbial stimulations. 2. Identification of 17 novel genome-wide significant cytokine QTLs (cQTLs) 3.cQTLs are enriched for regions under positive selection 4. Most cQTLs overlap with loci associated with infectious and autoimmune diseases.
Schirmer et. al (2016)	Human gut microbiome (Taxonomic and functional profiling)	1.Correlating cytokine responses with gut microbial profiles showed three interaction patterns: stimulus-specific, cytokine-specific and cytokine and stimulus specific associations 2. TNF-α and IFN-γ production are correlated with metabolic pathways, specifically palmitoleic acid metabolism and tryptophan degradation to tryptophol
Oosting et al. (2016)	Non-genetic (age, sex, tick bites, smoking, vitamin D) and genetic factors on Borrelia-induced cytokine response	1. Age strongly impaired IL-22 responses but no effect on IL-17 levels 2. HIF-1α-mediated glycolysis plays important role in regulating cytokine response to Borrelia. 3. HIF-1α pathway activation and increase in glycolysis-derived lactate was also confirmed in Lyme disease patients 4.Two genome-wide significant cQTLs: rs17615278 and rs11103976 associated with IFN-γ were identified
Bakker et al. (2018)	**Host factors** (such as age, sex, cell counts, metabolites, season, platelets, modulators, hormones and immunoglobins) **Molecular profiles** (genome, metabolome, gut microbiome) **Baseline immunological parameters** (IL-18, IL-18BP, resistin, leptin, adiponectin and α-1 antitrypsin)	1. Host factors explain up to 67% variation in cytokine levels 2. Genetics contributes greatly to interindividual variation (average adjusted R^2^ = 0.18) in cytokine response to pathogens. 3. Individuals with high genetic risk of (auto)immune disease tend to be high producers of cytokines in response to pathogens

## 4 Application of human functional genomics approaches to other traits than cytokine responses

Complementary to cytokines, other molecular phenotypes have been studied to boost our knowledge on the factors contributing to inter-individual immune response heterogeneity to microbial challenges. For example, Piasecka et al. (2018) recently examined the impact of age, sex and genetic factors on the inter-personal differences in transcriptional response to infection among 1000 individuals of European ancestry matched for age and sex [24]. The researchers demonstrate that age and sex influence the expression levels of most immune-related genes. Relative to sex effects, age effects tend to be more stimulus specific, which were largely shared across stimulations. Also, eQTL analyses revealed that genetic factors exhibit stronger effect on the expression of limited number of immune genes than age and sex. Furthermore, most detected *trans*-eQTLs had stronger effect upon stimulation, particularly *Candida albicans*-specific master regulator at the *CRI* locus, indicating that most of the identified trans variants in the *CRI* (a receptor for C3b and C4b split products) locus are related to immune response to *C. albicans*. This finding pinpoints stimulus-specificity of genetic variants regulatory effect on immune response.

In addition to transcriptomics and cytokine responses, metabolites also provide a crucial information to understand disease mechanisms. For example, in a study to identify specific biological pathways relevant to tuberculosis meningitis outcome in an Indonesian cohort, van Laarhoven et al. (2018) applied functional genomics approach, and measured complete cerebrospinal fluid (CSF) and serum metabolome in HIV-uninfected tuberculosis meningitis patients and controls [25]. In this study, CSF tryptophan concentrations were 9-times lower in patients who survived compared with patients who died. In a second cohort, they validated these findings and showed that low CSF tryptophan predicted patient survival. Furthermore, by performing a genetic study to identify loci predicting CSF tryptophan concentrations, they identified 11 of tryptophan QTLs. These QTLs predicted survival in a third cohort of tuberculosis meningitis patients.

In another study, Quach et al. (2016) leveraged RNA sequencing (RNA-Seq) to characterize the responses of primary monocytes, from individuals of European and African origin, to diverse Toll-like receptor (TLR) ligands and influenza A virus subsequent to eQTL mapping. The researchers identified not only numerous cis-eQTLs contributing to differences in immune response both within and between populations, but most importantly, a strong trans-eQTL hotspot at TRL1 that decreases expression of proinflammatory genes in Europeans only [26]. This study reveals that the determinants of differences in host immune responsiveness between human populations are largely distinct. Yet, to this end, most of the studies performed so far under the HFGPs have focused on individuals of European ancestry especially in the context of cytokines. However, the immune response to pathogens varies substantially among populations as multiple factors, such as genetics, epigenetics, (post)transcriptional and (post)translational regulation, lead to inter-person and interpopulation variations [27]. Besides, it is well known that genetic architecture of ethnically diverse groups is highly heterogenous due to population specific variation and variations in allele frequency. The varying differences in genetic architecture among populations can influence or can lead to differential effect on people's response to drugs and its adverse effects. For instance, common variants in *CYP2C9, VKORC1, and CYP4F2* have been reported to account for up to 18%,30%, and 11% respectively, of the variance in stable warfarin (anticoagulant) dose among patients of European ancestry but these variants explain less of the dose variability in patients of other ancestries [28]. For a safer, reliable and quality medical care for all, functional genomics studies across diverse populations is indispensable. Table 2 summarizes the content of the afore-mentioned studies.

**Table 2:** Summary of studies under HFGPs interrogating traits variability excluding cytokines.

Author (Year)	Factor(s) analyzed	Population	Main Findings
Piasecka et al. (2018)	Age, sex, genetic factors and gene expression	Healthy European cohort	1. Compared to sex effects, age showed more stimulus-specific effect on transcriptional response of most immune-related Genes 2. eQTL analyses revealed that genetic factors had a stronger effect on immune gene regulation than sex and age 3. Immune response eQTLs are organized in stimulus-specific manner
Laarhoven et al. (2018)	Cerebrospinal fluid (CSF), serum metabolites and genetics	Indonesian cohort of patients with Tuberculosis meningitis and controls	1. CSF tryptophan concentrations were 9- times lower in patients who survived compared with patients who died. 2. 11 genetic loci predictive for CSF tryptophan concentrations in tuberculous meningitis were identified 3. Cerebral tryptophan metabolism is important for the outcome of tuberculous meningitis
Quach et al. (2016)	Genetics and gene expression	Africans and Europeans	1. *Cis*- and *trans*-eQTLs contribute to population difference in immune responses. 2. Identified trans-eQTL hotspot at *TLR1* that decreases expression of pro-inflammatory genes in Europeans only. 3. Neanderthals introduced variants affecting immune responses into European genomes.

## 5 The need for population-specific human functional genomics projects

The desire to perform comprehensive functional genomics studies across diverse populations has in part, strongly motivated by findings of GWAS. GWAS have led to the discovery of more than 300 susceptibility loci for autoimmune diseases [29]. Although more than 2800 human GWAS are recently catalogued, studies that investigated the susceptibility to infectious diseases or the response to therapies for the treatment and prevention of infections are just a small fraction of these (4% and 0.66% respectively) [30]. Furthermore, approximately 80% of subjects in the GWAS catalog, and in most of the studies on the genetic association with disease were primarily of European descent [31]. The under-representation of non-European studies impedes our ability to understand the genetic architecture of human diseases and to predict risk score of diseases across general population. The poor transferability of genetic findings from the European studies to African population is expected as we see clear difference in the genetic architecture between populations. For example, a study by Magalhães et al. (2012), generated genetic distance map by computing Euclidean genetic distance using SNPs from 51 worldwide human populations. Findings from this study further strengthens the need for population specific HFGPs where genetic similarity analyses show that compared to the European continental group, the African ethnicities show the greatest diversity. Also, hierarchical clustering of the 51 populations showed two main blocks with distinct separation between African and non-African populations [32]. Below, we will discuss some of the studies that compared the utility of genetic findings across different populations.

In the context of assessing the transferability of genetic findings to other populations, a study was conducted to investigate how key genetic variants implicated in gene expression differ among populations and subsequent effects on genetic prediction. By performing eQTL mapping in individuals with African American, Hispanic, and European ancestry, the researchers demonstrated that the genetic architecture of gene expression for most predictable genes is sparse [33]. There were genes, for example *TACSTD2* that are well predicted, while others are poorly predicted across populations. Another interesting insight revealed from this study is that some genes that are well predicted in one population, predicted abysmally in other populations, demonstrating the need for comprehensive genomic studies in diverse population. In the same context, RNA-seq-based immune response eQTL study to investigate the transcriptional response to various live bacterial pathogens effects on African versus European ancestry individuals, reported markedly stronger response to infection induced in macrophages from individuals of African descent, with inflammatory response genes being more pronounced [34].

Another comparative study of variation in the transcriptional response of peripheral blood mononuclear cells to bacterial and viral stimuli between Batwa rainforest hunter-gathers and Bakiga agriculturalist from Uganda demonstrate that positive natural selection has contributed to shaping population differences in immune regulation. Importantly, across the set of genetic variants driving inter-population immune response differences, the signatures of positive selection were disproportionally observed in the rainforest hunter-gathers [35]. These findings emphasize the relevance of inter-population genetic analysis and also support the argument for intrapopulation analysis especially in Africans.

In addition to transcriptional response studies that we discussed above; many studies also tested the utility of polygenic risk score from one population to predict the phenotype in another population. For example, a study by Martin et al. (2017) utilized published summary statistics to construct polygenic risk scores for eight well-studied phenotypes to define the role of demography on polygenic risk prediction derived from a single-ancestry GWAS. They found that polygenic risk scores derived from European GWAS are biased when extrapolated to divergent populations even when the same causal variants are used, with directional inconsistencies in all scores. For example, despite anthropological evidence suggesting that West Africans on average are as tall as Europeans, height was predicted to decrease with genetic distance from Europeans. Most intriguingly, polygenic risk scores computed for type II diabetes across populations was observed to vary when summary statistics are derived from either Europeans-specific or multi-ethnic cohorts [36].

In addition to genetic background, of course the environmental challenges such as food habit, climate and pathogens also differ among these populations. Given the importance gene X environment interactions in determining complex phenotypes, there is a big gap in understanding the global clinical utility of genetic findings in one specific population.

**Figure 1:**
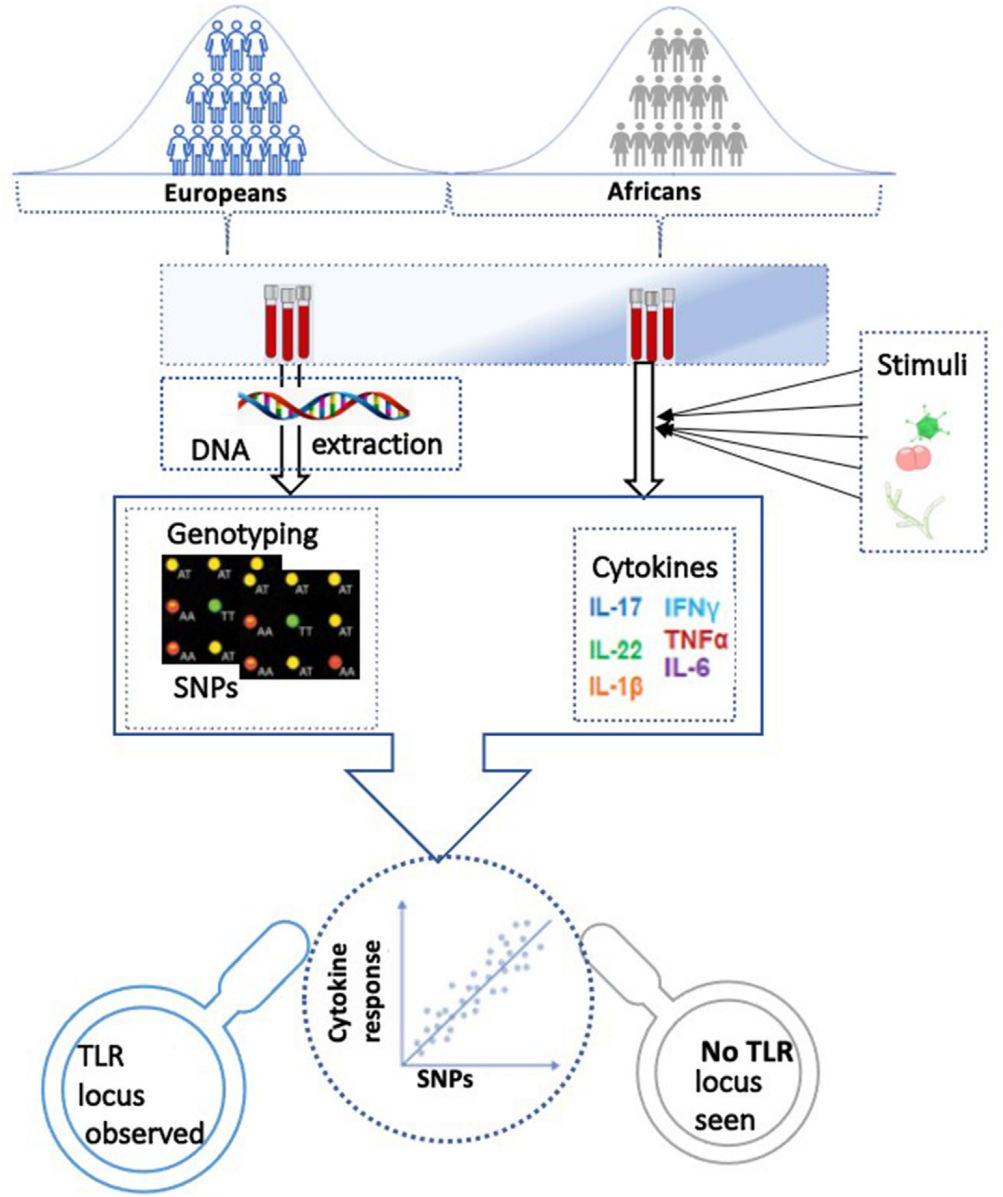
Schematic representation of the need for population-specific human functional immune-genomics projects. Cytokine quantitative trait loci (cQTL) analyses integrating genome-wide SNPs and induced-cytokine responses identified the TLR locus to be associated with cytokine response in individuals of European descent only.

To bridge this knowledge gap, the first of the series of HFGPs to be conducted in non-European populations is beginning to yield interesting findings. Some genetic variants have been identified to be associated with induced cytokine responses upon stimulations with pathogens using European cohort within the HFGPs. Already, interesting genes, pathways and useful insights have been uncovered from such studies. For example, a previous study showed that the TLR locus is associated with cytokine production capacity for many stimulations [13]. Strikingly, a comparable ongoing study using individuals of African descent failed to identify this locus to be associated with induced cytokine response (Figure 1). As a result, it is expected that analyses on genetic variations among diverse populations will help to identify additional loci, understand further the mechanisms underlying differential response of the immune system.

## 6 Future perspectives

**Figure 2:**
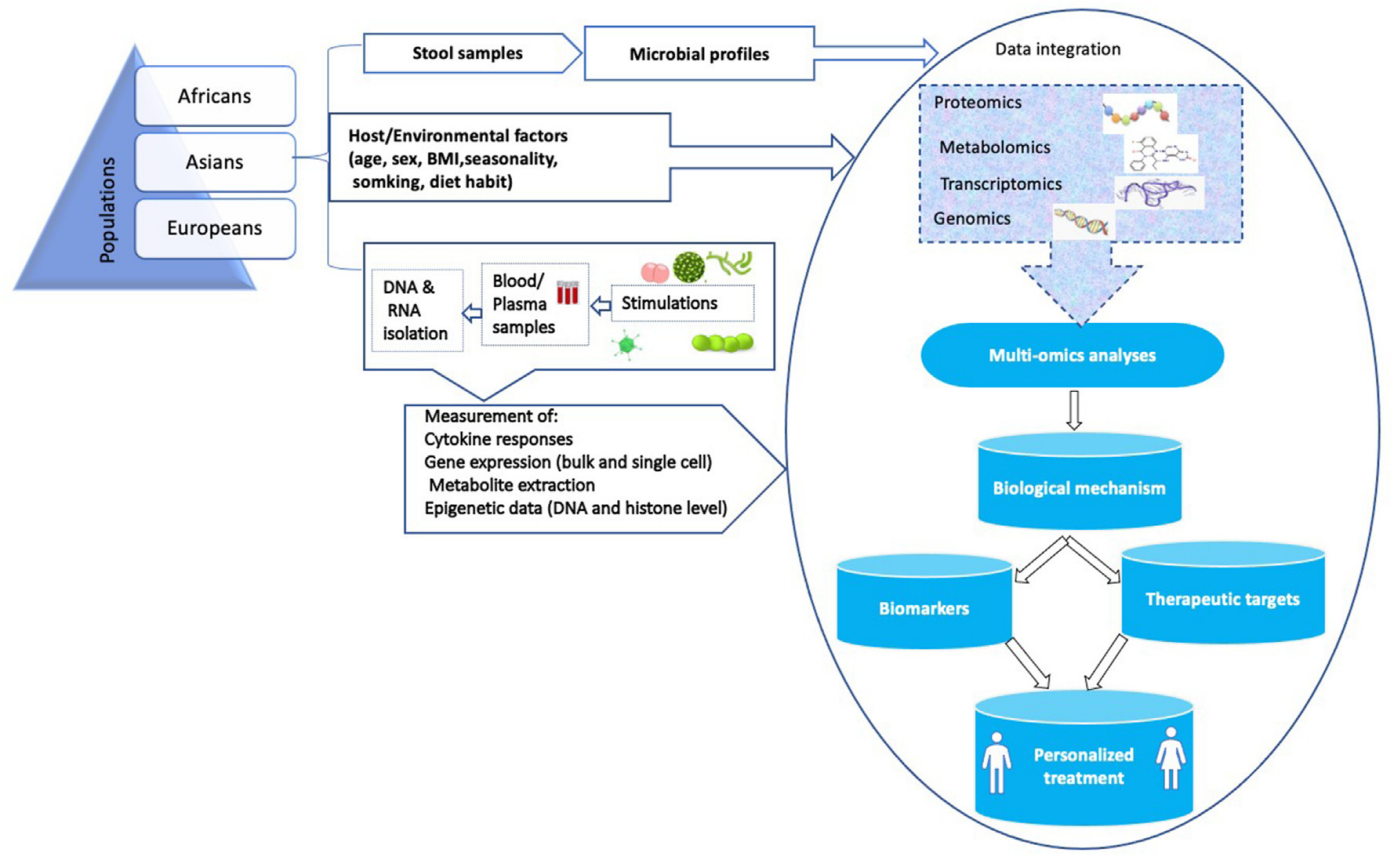
Schematic overview of the concept of human functional immune-genomics projects. The concept aims at recruiting individuals of diverse human populations and generating large population-based cohorts. Among other information gathered from the individuals are metadata, stool samples, plasma samples, microbial samples and DNA for genomics studies. Advanced multi-omics approaches are used to integrate and analyze various datasets with subsequent identification of biomarkers and therapeutic targets to understand how inter-individual and inter-population immune response variation underlies disease mechanisms. Characterizing the factors driving immune response variation is critical to the realization of the concept of personalized treatment.

Taken together, whilst significant progress has been achieved since the advent of the HFGPs, it is important to highlight the areas yet to be explored. To this end, all analyses performed focused primarily on genomics, metabolomics and added a limited amount of transcriptomics to validate research findings or prioritize candidate genes. However, to better understand the basis of varying immune responses in individuals, these single ‘omics’ approaches will not be sufficient given the complexity of the biological system. Therefore, future HFGPs seeks to adopt integrated omics analyses, the current trend to achieve clear mechanistic insights into the basis for differences in human cytokine responses among individuals, and the wide range of phenotypic variation observed across human populations (Figure 2). In this context, few studies have been performed. Recently, a study using European cohorts only integrated genomic, metagenomic, metabolic data, immune cell composition and platelet activation profiles from individuals [23]. Findings by integrating multi-omics layers show that cytokine production is regulated by both multiple genetic and non-genetic host factors. Strikingly, they reported that cytokine responses induced by stimulations could be moderately predicted using multiple baseline profiles. By leveraging this approach, the authors identified new modulators of cytokine production. Circulating metabolites, among which acetate, were associated with changes in stimulus-induced cytokine production and especially in the modulation of T_H_1 and T_H_17 responses. Also, volunteers with increased genetic risk for immune-mediated diseases were more likely to be high responders in terms of stimulus-induced cytokine production.

Additionally, applying various omics technologies such as genomics, proteomics, metabolomics and transcriptomics at single cell level has become the current routine when investigating cell-to-cell heterogeneity [37]. Recently, de Vries et al. (2020) integrated genome-wide association studies with bulk and single cell transcriptomics analyses of immune cells stimulated with *C. albicans* to deepen our understanding of anti-Candida host response. By leveraging multi-omics technique and cell-type dependent differential analysis, they show that important cell types such as monocytes and natural killer (NK) cells are involved in host response against *Candida* [38]. These findings provide not only important insight into disease biology but also serve as a framework for future studies to extend this analysis with various pathogens in different populations.

Another important area to tackle is longitudinal genetic studies. The common approach of investigating the effect of genetic variants at individual time points (e.g. SNP at a time) on phenotypic variation has revealed some important insights. However, this method may not reveal variants that are affected by epigenetic modifications in different cell types and tissues over time. The inclusion of repeated measurements provides additional advantage among others, in understanding the trajectory of traits and disease progression [39]. Therefore, longitudinal data collection and analyses across diverse populations is imperative to broaden our knowledge on immune responses to pathogens over time and to answer questions such as which genetic variants influence inter-individual variability independent of epigenetic modifications over time.

## 7 Conclusion

Even though the advent of the HFGPs had an unprecedented impact on our knowledge, bringing into light the genetics and non-genetic host factors contributing to inter-individual heterogeneity in immune responses, the expansion of such studies to incorporate diverse populations is needed to realize the personalized medicine in every population. While most of immunogenetic (e.g. cQTL mappings) and other studies have been performed in populations of European descent, increasing the number of studies in non-European populations (using both patient and population-based cohorts) will make the necessary comparisons between populations feasible. In addition, this is particularly important considering the fact that findings from studies using European cohorts within the HFGPs is not easily transferable, replicating these findings in different populations will help to pin-point population-specific variants and bridge the existing disparities in genetic knowledge for non-European populations. In addition, the reductionist approach to investigating the basis of variability in cytokine responses may obscure important insights. In future HFGPs focused on the immune responses to pathogens, we envision that integrative approaches to studies of ethnically diverse human populations will contribute to holistically examine the main and large-scale interactions of different omics layers to advance our knowledge and understanding of health and disease.

## Declarations

### Author contribution statement

All authors listed have significantly contributed to the development and the writing of this article.

### Funding statement

This work was supported by a Hypatia tenure track grant (10.13039/501100006209RadboudUMC) and a Research Grant [2017] of the European Society of Clinical Microbiology and Infectious Diseases (10.13039/501100001704ESCMID) to V.K, European Research Council (10.13039/100010663ERC) Consolidator Grant [FP/2007–2013/ERC grant 2012–310372] and a Netherlands Organization for Scientific Research (10.13039/501100003246NWO) Spinoza prize grant [NWOSPI94-212] to M.G.N.

### Data availability statement

Data will be made available on request.

### Declaration of interests statement

The authors declare no conflict of interest.

### Additional information

No additional information is available for this paper.
